# CATH: comprehensive structural and functional annotations for genome sequences

**DOI:** 10.1093/nar/gku947

**Published:** 2014-10-27

**Authors:** Ian Sillitoe, Tony E. Lewis, Alison Cuff, Sayoni Das, Paul Ashford, Natalie L. Dawson, Nicholas Furnham, Roman A. Laskowski, David Lee, Jonathan G. Lees, Sonja Lehtinen, Romain A. Studer, Janet Thornton, Christine A. Orengo

**Affiliations:** 1 Institute of Structural and Molecular Biology, UCL, 636 Darwin Building, Gower Street, WC1E 6BT, UK; 2 London School of Hygiene and Tropical Medicine, Keppel Street, London, WC1E 7HT, UK; 3 European Bioinformatics Institute, Wellcome Trust Genome Campus, Hinxton, Cambridgeshire, CB10 1SD, UK

## Abstract

The latest version of the CATH-Gene3D protein structure classification database (4.0, http://www.cathdb.info) provides annotations for over 235 000 protein domain structures and includes 25 million domain predictions. This article provides an update on the major developments in the 2 years since the last publication in this journal including: significant improvements to the predictive power of our functional families (FunFams); the release of our ‘current’ putative domain assignments (CATH-B); a new, strictly non-redundant data set of CATH domains suitable for homology benchmarking experiments (CATH-40) and a number of improvements to the web pages.

## INTRODUCTION

Next generation sequencing continues to flood bioinformatics databases with new protein sequences, most of which are of unknown function. Being able to suggest the roles that these proteins play and the likely mechanisms by which they play them from protein sequence alone would have hugely important implications to many aspects of biological research. Making this kind of prediction requires a detailed knowledge of how protein function can change during the process of evolution and how these changes can be recognized from patterns within the protein sequence. Since a protein's function is intrinsically linked to its precise shape, 3D structure can often be used to detect very remote evolutionary relationships, even after the amino acid sequence has changed beyond recognition.

CATH (Class, Architecture, Topology, Homology) uses protein structure as a sensitive probe for remote evolutionary relationships, and also to provide an additional layer of insight into the relationship between sequence and function. CATH's sister resource, Gene3D, augments this by inheriting the knowledge gained from studying the ∼100 000 solved protein structures to the millions of known protein sequences. CATH-Gene3D's key roles in the coming years will be to harness the knowledge of sequence/structure/function relationships carefully constructed during nearly 20 years of expert curation to provide high quality functional annotations for all these new sequences.

CATH is a hierarchical protein domain classification. Protein structures are taken from the Protein Data Bank (PDB) and are chopped into individual structural domains. If there is sufficient evidence that a set of domains share a clear common ancestor, they are grouped together into a single homologous superfamily. When homologous superfamilies (‘H’ level in CATH) share the same fold, but do not have sufficient evidence to suggest a clear evolutionary relationship, they are placed in the same topology (‘T’ level). Topologies that share a roughly similar spatial arrangement of secondary structures are grouped in the same architecture (‘A’ level). The 40 architectures in CATH are arranged into one of four main classes (‘C’ level) based on the content of secondary structure (mainly alpha, mainly beta, mixed alpha-beta, few secondary structures).

Gene3D uses HMM technology to reliably match the sequences from CATH structural domains against the huge databases of protein sequences with no known structure. Since CATH domains are assigned to a superfamily in CATH, these evolutionary relationships can also be inherited to the predicted domains. Gene3D also imports a variety of functional annotations from a number of different public bioinformatics resources. Doing so considerably augments the knowledge of sequence diversity and functional annotations that can be associated with CATH superfamilies.

### Hierarchy versus fold continuum

As more protein structures have been solved, it has been observed that in some cases it can be unclear where one fold ends and another begins (1). This observation suggests that a ‘fold continuum’ model may be more appropriate than a strict hierarchy. While our own research supports this hypothesis, we find the scope of the issue is limited to certain areas of structural space, hence we have retained the C, A and T levels of CATH to help organize our homologous superfamilies into general regions of structural space. We represent this fold continuum by including a list of ‘lateral links’ that identify nodes of the hierarchy with structural relationships that do not fit neatly within this hierarchical model.

### Functional families

Homologous superfamilies in CATH can incorporate a great deal of structural and functional diversity (2). However, when predicting function, it is important to recognize the precise sequence patterns that can be associated with a particular functional role. To achieve this, we must first attempt to isolate the sequences associated with that function.

To this end, all the predicted domain sequences assigned to CATH superfamilies have been subclassified into functional families (FunFams). Relatives within these functional families are likely to share highly similar structures and functions. In our latest major release of CATH we have improved our subclassification protocol to provide more accurate functional families. We have also significantly expanded the resource and added some valuable new features described below.

## CATH 4.0 RELEASE HIGHLIGHTS

### New domain assignments

The latest version of CATH (v4.0) adds annotations for over 62 000 new structural CATH domains and over 100 new superfamilies. This increase was enabled by a number of (carefully benchmarked) improvements to the automated assignments made in the CATH update workflow. It also represents a significant investment of time in expert curation to resolve the more complex cases of domain chopping and homology assignments.

### CATH-B

The process of creating an official release of CATH involves many steps: cross-checking to identify additional homologies, updating sequence assignments and alignments, adding layers of data, etc. All of these steps are important for providing consistent and scientifically useful information, however, this release process is also responsible for a delay between structures being made available in the PDB and their assignments made available in CATH. For example, there are now over 235 000 fully assigned CATH domains in the latest official release of CATH (4.0). However, at the time of writing, the number of fully assigned domains in our working version of the database is just under 300 000.

‘CATH-B’ provides access to putative annotations to help bridge the gap between the bleeding-edge PDB release and the most recent official CATH release. Currently, these annotations are available as downloadable files, provided at http://release.cathdb.info/cath_b/.

These files are updated daily and reflect the latest manual and automated assignments in our database. Though it is possible that these annotations may change when we provide an official release of CATH, in practice we expect changes to these assignments to be rare and mostly due to extra evolutionary relationships being identified rather than existing relationships being removed (i.e. two superfamilies may be merged due to new evidence linking them together).

### CATH FunFams

CATH-Gene3D provides domain predictions and superfamily assignments for protein sequences in UniProt. All domains (both ‘real’ and predicted) within a CATH superfamily are then classified into FunFams using a hierarchical agglomerative clustering algorithm. The algorithm produces a tree of clusters and this was originally partitioned using a generic threshold (3). However, this partitioning was later improved by using functional annotation data from the Gene Ontology (GO) (4) to ensure functional coherence (5). Significant biases in GO annotations have recently been identified (6) suggesting that GO annotation data can only provide a partial picture of the function of a protein, which will affect functional classification.

The FunFams in CATH v4.0 have been identified by using a new family identification protocol: FunFHMMer. This algorithm recognizes evolutionary signals in cluster alignments, such as highly conserved positions (7) and specificity-determining positions (8), and uses this information to ensure functional coherence (manuscript under preparation). The FunFams have been further associated with GO terms probabilistically, in order to provide function annotations for uncharacterized sequences.

**Figure 1.**
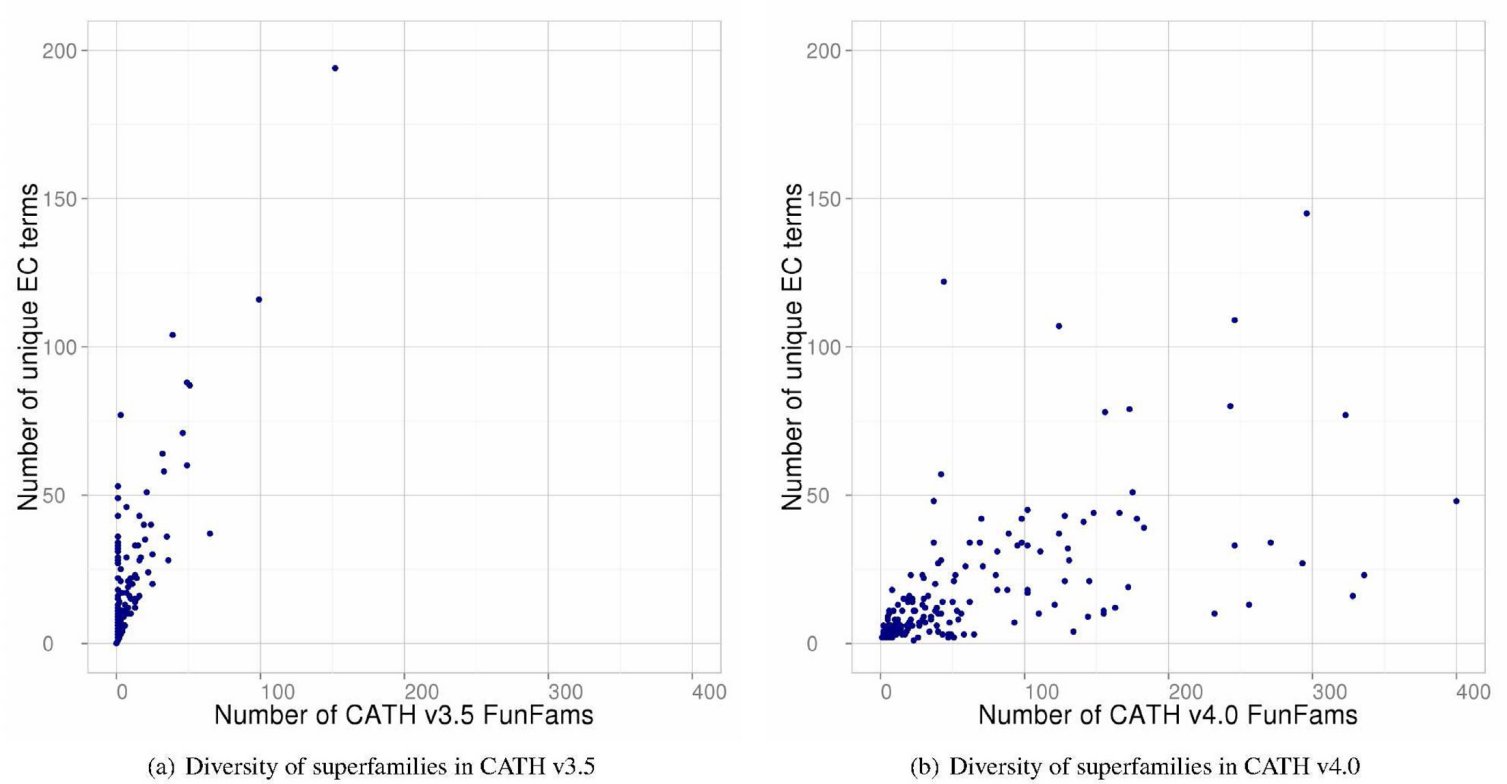
Comparison of EC diversity against the number of FunFams for superfamilies in CATH v3.5 and v4.0.

A comparison of the Enzyme Commission (EC) (9) number diversity against the number of FunFams identified for 200 CATH enzyme superfamilies containing catalytic domains (Figure 1) clearly shows the improvement in the functional classification in the latest release. Thirty superfamilies containing more than 10 unique EC numbers have been classified into one FunFam in CATH v3.5. By contrast, only four superfamilies in CATH v4.0 have been grouped into one FunFam which contains two unique EC numbers. Looking closely at one large functionally diverse enzyme superfamily, it becomes apparent that the functional classification in CATH v4.0 provides more functionally coherent families than those in CATH v3.5.

**Figure 2.**
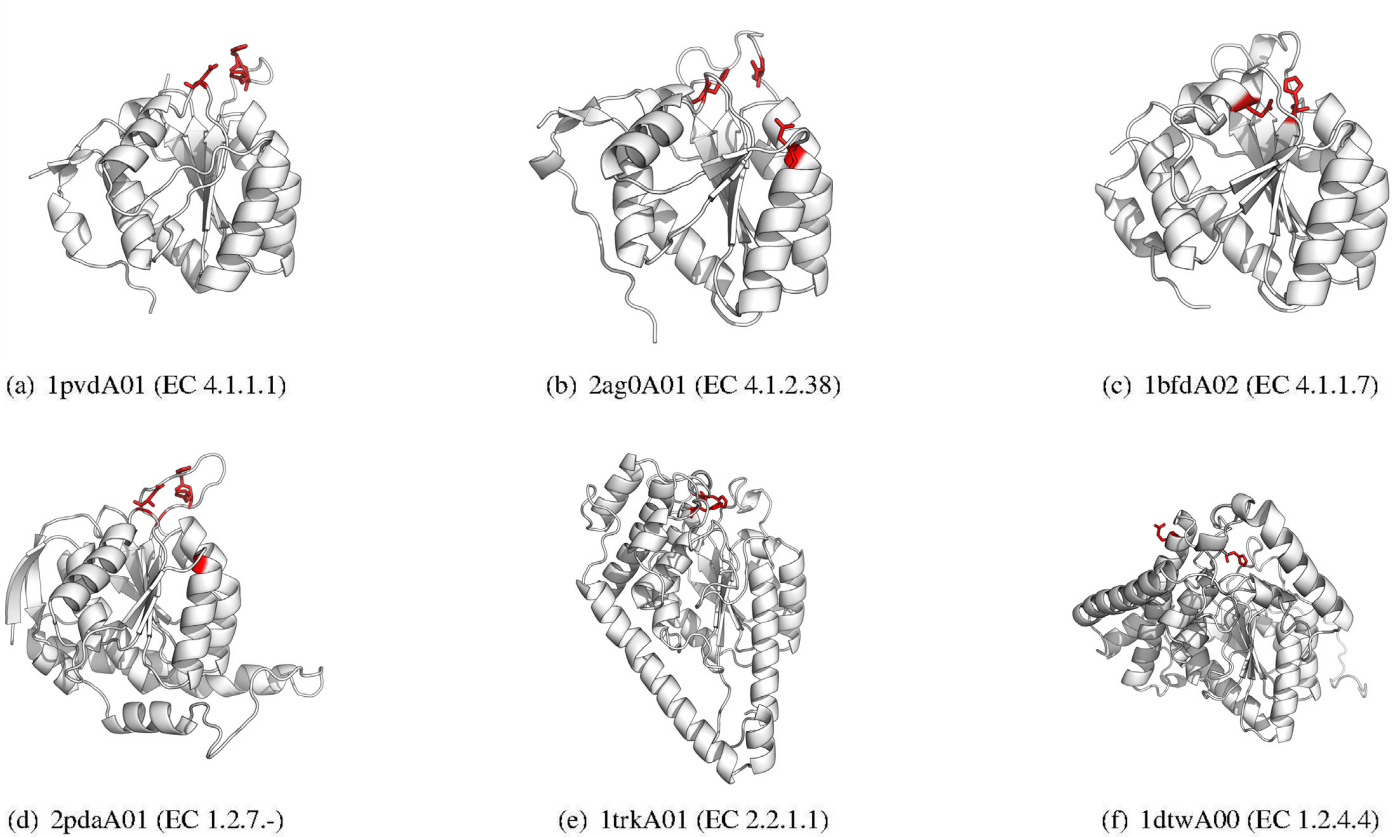
Selected TPP-dependent enzyme superfamily (CATH 3.40.50.970) domains classified into different FunFams in CATH v4.0 using the improved FunFHMMer algorithm. The previous version of CATH (v3.5) clustered all members of this superfamily into one, large FunFam. The catalytic site residues derived from the CSA (Catalytic Site Atlas) are highlighted in red and shown as sticks.

For example, the well-studied Thiamine pyrophosphate (TPP)-dependent enzyme superfamily (10) (CATH superfamily: 3.40.50.970), which contains 89 unique EC numbers, has been classified into 140 FunFams in CATH v4.0 compared to a single, large FunFam cluster in CATH v3.5. Figure 2 shows a few selected TPP-dependent enzyme superfamily domains classified in different FunFams in v4.0 which all perform very different functions.

Further validation of our new functional subclassification protocol comes from the preliminary results of the recent CAFA international function prediction experiment which ranked FunFHMMer fourth out of the 110 methods for ‘molecular function’ prediction and second for ‘biological process’ prediction. FunFHMMer was the top domain-based method (P. Radivojac, personal communication).

Since the functional profiles of the improved FunFams in CATH v4.0 are domain-based, they are expected to give improved coverage in recognizing protein functions of multi-domain proteins compared to resources based on whole proteins. In addition, the ability to assign uncharacterized sequences to FunFams and obtain information on conserved functional sites will be important for understanding the consequence of residue mutations (e.g. single-nucleotide polymorphisms (SNPs)) in genetic variants of these proteins.

## NEW DATA IN CATH

The latest release of CATH provides a number of new data types available for download which we think will benefit the community.

### CATH-40

Previous versions of CATH have provided lists of representative domains, with each domain representing a cluster of domains that are all guaranteed to share a minimum sequence identity. Each homologous superfamily has one or more sequence family cluster(s) (‘S’ level) and every domain in the same ‘S’ cluster shares at least 35% sequence identity. This strategy is useful when trying to choose a single domain that best represents every other structure in that cluster. However, since this method is not able to guarantee that two domains in different clusters are not related (i.e. domains in different ‘S’ clusters may share more than 35% sequence identity), this list is not as well suited for use as a non-redundant data set (e.g. to benchmark algorithms predicting remote homology).

To address this issue, CATH now provides an additional data set of non-redundant CATH domains. This is designed to be the largest list of domains that we were able to generate while ensuring that Basic Local Alignment Search Tool (BLAST) (11) is not able to identify any pair of domains that share at least 40% sequence identity over at least 60% overlap. This can be considered analogous to the ASTRAL40 data set (based on the SCOP classification) (12).

To implement this, we begin with a list of representative domains from the existing clustering at 100% sequence identity. We then perform an all-versus-all BLAST of these domains to identify pairs that meet the 40%/60% criteria described above. These data are then fed into an algorithm that attempts to build the largest possible list of representatives with no such links between any two members. The algorithm begins with an empty list of representatives and then iteratively adds members. At each step, it chooses a domain with the fewest linked neighbours in an attempt to nibble as many edges off a cluster as possible, rather than choosing a small number of well-connected domains at the cluster's centre.

The data set is available as domain identifiers, sequences in FASTA format (optionally restricted to residue in the PDB files) and PDB files. This can be found at: http://release.cathdb.info/latest_release.

### Superfamily superpositions

**Figure 3.**
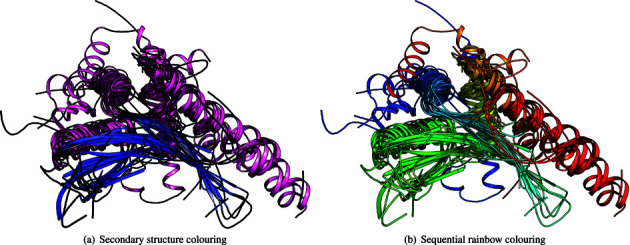
Superposition of 15 representative domains from the Homing endonucleases superfamily (CATH: 3.10.28.10).

Since the previous paper, we have added superfamily superpositions to the CATH superfamily web pages. These are superpositions of all representative domains within a superfamily (selected from sequence clusters at 35% sequence identity over 80% overlap). Figure 3 shows a superposition of the 15 representative domains in the Homing endonucleases superfamily in CATH v4.0 (3.10.28.10) coloured by secondary structure and by rainbow.

These superpositions are generated by combining pairwise alignments provided by the SSAP algorithm (13). The pairs are selected by constructing a maximal spanning tree from a full, all-versus-all matrix of pairwise SSAP scores. Importantly, the superposition algorithm attempts to prevent divergent regions from disrupting the superposition of structurally similar regions. The benefit of this is most pronounced in cases with extremely divergent regions but it also tightens superpositions of more consistent groups. The algorithm achieves this effect by exploiting the SSAP residue-pair scores for each position in the alignment to identify structurally divergent positions and then disregarding these positions when superposing the structures (14).

Each superposition is presented on the corresponding superfamily web page as a static image and as a downloadable PyMOL script. Images are provided that are coloured either by secondary structure or with a sequential rainbow gradient. The rainbow colouring is performed over the alignment, rather than over each individual structure, meaning that residues identified as structurally equivalent are assigned the same colour. Further, the scores for the positions of the alignment are used to determine the rate of change of the gradient so that it changes little over the divergent regions, leaving the majority of the spectrum for the more conserved regions.

For many superfamilies, these superpositions provide powerful illustrations of how well the core can be conserved throughout evolution despite the broad diversity exhibited in overall structure.

### HMM libraries

A number of CATH users have expressed an interest in running their own local sequence searches against the HMM libraries used in CATH. We now provide access to a library of HMMs generated using the jackhmmer utility (HMMER3, (15)), seeded from representative domains in CATH. We also provide access to the library of HMMs built from the sequence alignments that represent our FunFams. Both of these libraries can be downloaded via http://release.cathdb.info/v4.0.0/hmmer3/.

### FunTree

When investigating protein evolution it is important to consider structural domains as independent components. However, it is also important to consider how function can evolve through interactions between different combinations of domains within a protein. To provide a view of the multi-domain organization of each CATH domain superfamily, an interactive force-directed graph using the domain assignments from CATH-Gene3D has been implemented using the D3 JavaScript library. The graph connectivity is calculated using the ArchSchema algorithm (16) based on sequences found in the reviewed section of UniProt (17). At each node, domains with associated structural data are listed and linked to PDBe (18) as well as EC numbers if any sequences with enzyme function share the node's domain architecture.

The structural cluster alignments generated from CATH v4.0 have been subjected to the FunTree pipeline (19). Preliminary data for 1926 CATH superfamilies are available at http://cpmb.lshtm.ac.uk/FunTree as a beta release. For each structural cluster within a superfamily, a phylogenetic tree is generated based on a filtered alignment. The representatives in the filtered alignment are chosen to maximize functional and taxonomic diversity, while maintaining as many sequences as possible with a solved structure.

### Improvements to the web pages

The CATH web pages are continually being updated and improved, often in response to feedback from users. A number of significant improvements have been made since the last updated article.

#### Removing Java dependencies

A number of platforms do not support Java (especially mobile platforms). In addition, the browsers running on platforms that do support Java often have default security settings that block the use of applets. We have moved the tools and components that were dependent on Java over to solutions based on Javascript/HTML5.

#### Incorporating BioJS components

BioJS is a rapidly growing, community-driven project that aims to help users and developers represent biological data on the web (http://biojs.net). We have been active members of this community: we have already incorporated BioJS tools into the CATH web site and will continue to do so (and contribute any features and improvements back to the BioJS community).

#### Browsing CATH

**Figure 4.**
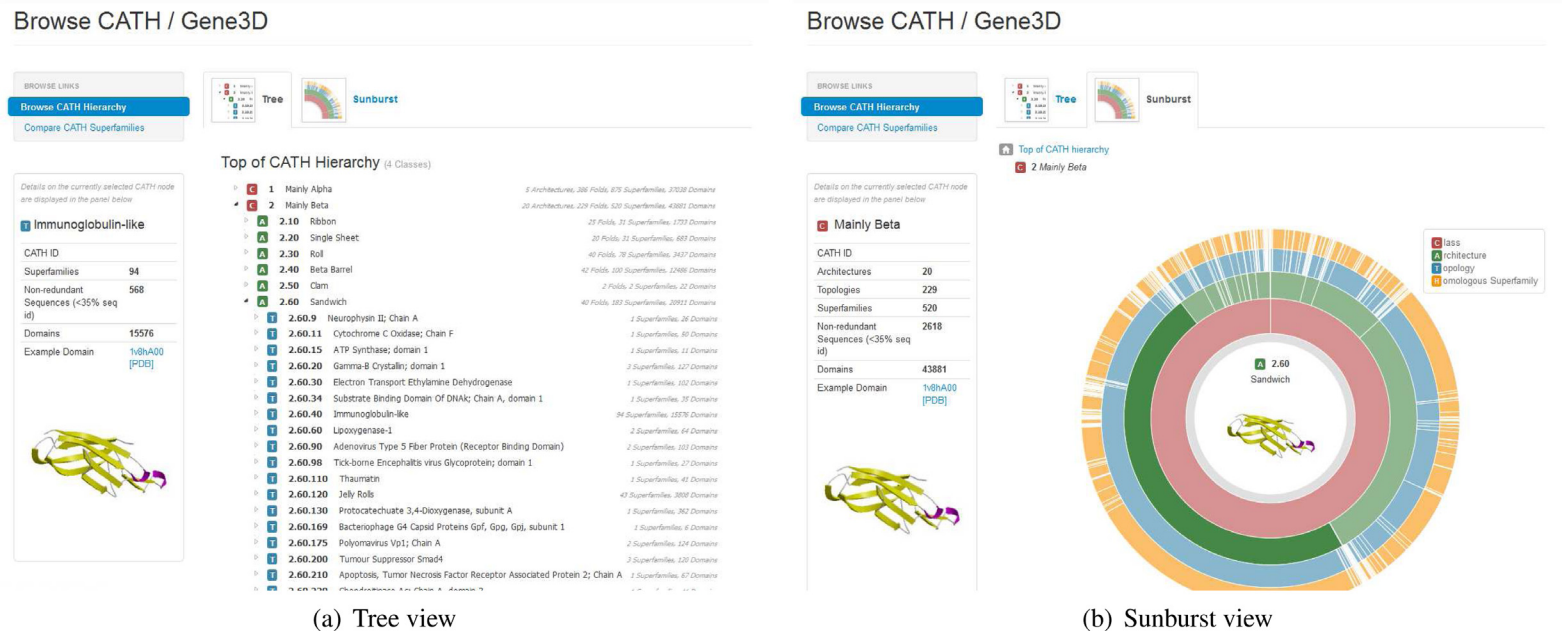
New tools allow users to browse the CATH structural hierarchy using dynamically generated views: ‘Tree’ and ‘Sunburst’.

Users have informed us that our web pages are often used to help teach students about the universe of known protein structures. We now provide tools to help users to browse through the different types of structures in CATH quickly and easily (see Figure 4).

## CONCLUSION

This paper announces the release of CATH-Gene3D v4.0, which contains over 235 000 structural domains and 25 million sequence domain predictions. It also describes our work to expand and improve the data we associate with CATH-Gene3D to make it more powerful and useful to the community.

We have investigated ways to improve how we subgroup the sequences assigned to a superfamily into FunFams. This has resulted in FunFHMMer, a new protocol that recognizes evolutionary signals in cluster alignments, such as highly conserved positions and specificity-determining positions. This protocol has been used to generate the v4.0 FunFams and we have found that these exhibit better functional coherence than those generated with the previous release. The FunFHMMer protocol performed excellently in the recent CAFA international function prediction experiment.

From v4.0 onwards, we will provide the community with CATH-B, a daily update on the domain assignments that have been performed since the latest full release. Though these CATH-B assignments should not be considered gold standard, in practice, we expect them to undergo few changes before being fully released, other than those arising from new homologies being identified between superfamilies.

With v4.0, we have added new types of data to accompany CATH releases. We now provide CATH-40, a convenient, strictly non-redundant data set of CATH domains. This should not contain any two domains that share more than 40% sequence identity over 60% overlap according to BLAST, making it well-suited to benchmarking. For each superfamily in CATH v4.0, we now provide a superposition of all the representatives in the superfamily (at 35% sequence identity), both as an image and as a downloadable PyMOL script. With v4.0, we now also provide access to the HMM libraries that we use to identify sequence relationships.

The FunTree pages provide a powerful interface to explore the multi-domain organization within CATH superfamilies and to provide insights into how this affects the evolution of function.

Combined with the rapid growth of the CATH-Gene3D data, these enhancements improve the accessibility of the resource, its usefulness to the community and its ability to provide insight into structure, sequence and function.
